# Limiting inbreeding in disjunct and isolated populations of a woody shrub

**DOI:** 10.1002/ece3.2322

**Published:** 2016-07-25

**Authors:** Jane F. Sampson, Margaret Byrne, Neil Gibson, Colin Yates

**Affiliations:** ^1^ Science and Conservation DivisionDepartment of Parks and WildlifeLocked Bag 104Bentley Delivery CentrePerthWestern Australia6983Australia

**Keywords:** Gene flow, inbreeding, isolation, mating system, paternity, pollen dispersal, self‐incompatibility

## Abstract

Pollen movements and mating patterns are key features that influence population genetic structure. When gene flow is low, small populations are prone to increased genetic drift and inbreeding, but naturally disjunct species may have features that reduce inbreeding and contribute to their persistence despite genetic isolation. Using microsatellite loci, we investigated outcrossing levels, family mating parameters, pollen dispersal, and spatial genetic structure in three populations of *Hakea oldfieldii*, a fire‐sensitive shrub with naturally disjunct, isolated populations prone to reduction in size and extinction following fires. We mapped and genotyped a sample of 102 plants from a large population, and all plants from two smaller populations (28 and 20 individuals), and genotyped 158–210 progeny from each population. We found high outcrossing despite the possibility of geitonogamous pollination, small amounts of biparental inbreeding, a limited number of successful pollen parents within populations, and significant correlated paternity. The number of pollen parents for each seed parent was moderate. There was low but significant spatial genetic structure up to 10 m around plants, but the majority of successful pollen came from outside this area including substantial proportions from distant plants within populations. Seed production varied among seven populations investigated but was not correlated with census population size. We suggest there may be a mechanism to prevent self‐pollination in *H. oldfieldii* and that high outcrossing and pollen dispersal within populations would promote genetic diversity among the relatively small amount of seed stored in the canopy. These features of the mating system would contribute to the persistence of genetically isolated populations prone to fluctuations in size.

## Introduction

Some plant species occur as disjunct populations separated from one another by unsuitable habitat over which migration and gene flow can be limited (Pannell and Fields 2014). Several studies suggest that even low levels of gene flow may be sufficient to maintain diversity within populations (Hoebee et al. 2007; Hsieh et al. 2013; Millar et al. 2013; Ellstrand 2014; Saro et al. 2014; George et al. 2015). But smaller, disjunct populations are more prone to reduced gene flow, and to increased genetic drift and inbreeding (Young et al. 1996). This can result in genetic impoverishment within populations, divergence among populations, and inbreeding depression, which can have important consequences for seed production and fitness (Ellstrand and Elam 1993; Coates et al. 2007). Within disjunct populations, inbreeding is likely to be increased because plants tend to mate locally (Pannell and Fields 2014).

The mating system is considered to be a key factor influencing the genetic diversity of plant populations (Pannell and Charlesworth 2000; Charlesworth 2006; Duminil et al. 2009). The dispersal of genes through pollen and seed is also important in determining population dynamics and viability because of its influence on local spatial genetic structure (Wells and Young 2002). Mating systems and gene dispersal are affected by the size, density, spatial genetic structure, and spatial separation of populations (Young et al. 1996; Sork et al. 1999; Hoebee et al. 2007), and also by individual plant characteristics such as plant size, floral display, flowering phenology, pollination, and self‐incompatibility (SI) systems (Barrett and Harder 1996; Richards 1997; Coates et al. 2007; Hoebee et al. 2007; Eckert et al. 2010).

Several reviews (Young et al. 1996; Honnay and Jacquemyn 2007; Wagenius et al. 2007; Aguilar et al. 2008; Eckert et al. 2010) have reported small population size and isolation to have negative effects on plant mating systems. But these reviews included relatively few studies of species with naturally disjunct population distributions. Several studies of plant species with disjunct distributions have found that genetic diversity is unrelated to census population sizes (Hoebee and Young 2001; Llorens et al. 2004; Leimu and Mutikainen 2005; Mathiasen et al. 2007; Sampson et al. 2015). Indeed, it has been suggested (Holmes et al. 2009; Hopper 2009) that naturally disjunct species may have ecological and genetic features that counter the negative effects of small size and isolation. For example, large seed banks can buffer against loss of diversity following population size reduction and inbreeding (Llorens et al. 2004); fitness can be maintained despite high selfing rates through purging of lethals and high seed production (Sampson et al. 2014); self‐incompatibility can promote diversity through multiple paternity in small, dense populations (Llaurens et al. 2008); longevity can permit outbreeding to be established over the long term (Hoebee et al. 2008); and some species may develop diverse patterns of genetic diversity through clonality and polyploidy (Holmes et al. 2009). Determining how mating systems within disjunct populations might maintain diversity despite small population size and genetic isolation requires information about seed production, outcrossing rates, spatial structure, and pollen movements.

*Hakea oldfieldii* Benth. (Proteaceae) is a dense multistemmed woody shrub that grows to 5 m and is found in naturally disjunct and isolated populations associated with the soils of uncommon isolated ironstone formations in the southwestern Australian biodiversity hot spot. The species is monecious and produces numerous axillary racemes with 8–20 hermaphrodite flowers generally between September and October. Low levels of fruit and seed production per flower are common within Proteaceae (Ayre and Whelan 1989; Harriss and Whelan 1993; Hoebee and Young 2001), and in *H. oldfieldii,* a small proportion of flowers successfully set woody fruit that are weakly serotinous (canopy‐stored seed). Fruit generally open in the second year and contain up to two large, heavy, winged seeds that are unlikely to disperse more than a few meters from the seed parent. The specific pollinators are not known, but field observations and floral morphology suggest pollination by insects rather than birds or mammals.

Populations are prone to fluctuations in size and extirpation because, although the species can be long‐lived (>50 years), plants are killed by fire that occurs frequently in the shrublands of southwestern Australia. A recent study (Sampson et al. 2015) suggested that low gene flow (*N*_m_ < 1.0), below the level required to reduce the harmful effects of local inbreeding (*N*_m_ > 1.0; Lowe and Allendorf 2010), genetic drift, and a demographic history of fluctuating population sizes including genetic bottlenecks have been the major determinants of the level and distribution of diversity in *H. oldfieldii*. Both long‐term historical and contemporary migration were very low (contemporary migration rate, *m *=* *0.001, long‐term *m *=* *0.003) among populations that show moderate levels of diversity and significant nuclear (microsatellite) and plastid divergence. Little is known of the mating system of *H. oldfieldii*. In the study of Sampson et al. (2015), inbreeding coefficients were positive (*F *=* *0.036–0.198) but did not indicate that inbreeding was an important component of the breeding system in 10 of 14 populations, and were not positively correlated with long‐term effective population size.

In this study, we investigated the outcrossing rate and spatial genetic structure in three populations of the woody shrub *H. oldfieldii*, and we used paternity analyses to reveal the extent of multiple paternity and pollen movements within populations. We hypothesize that disjunct and genetically isolated populations of *H. oldfieldii*, which rely on one or two seasons of canopy‐stored seeds to survive fires, will have high levels of outcrossing and extensive pollen dispersal to promote paternal and genetic diversity among seeds.

## Materials and Methods

### Study sites

**Figure 1:**
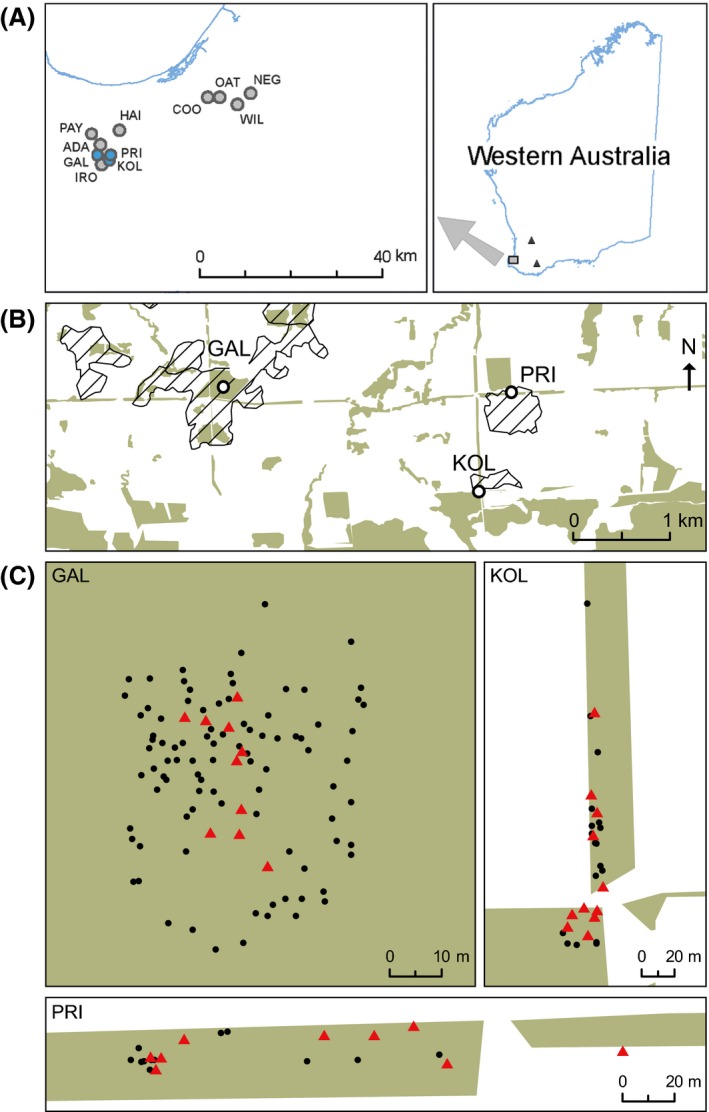
Locations of *Hakea oldfieldii* in southwestern Western Australia. (A) Regional distribution. Blue circles indicate study populations. (B, C) Diagrams of the three populations in which mating system and pollen movement studies were undertaken. Cross‐hatched areas represent ironstone soils. Green areas show areas of remnant vegetation. Solid circles indicate individual plants, and triangles indicate seed parents.

*Hakea oldfieldii* is known from three regions in Western Australia separated by between 130 and 280 km (Fig. 1). The center of the distribution is the winter‐wet (~800 mm) banded ironstone shrubland communities in the most western region. These communities are one of the most threatened vegetation types in Western Australia (Gibson et al. 2000), and therefore, sampling was limited to minimize the impact on populations.

Sampled populations were located in a 45 km × 15 km area in the western region that has been exhaustively surveyed for *H. oldfieldii*. The landscape in this area has been fragmented by the clearing of native vegetation for agriculture, mainly between 1901 and the mid‐1970s (Gibson et al. 2012). Therefore, to describe the ecological condition of populations, we used the classifications of Eckert et al. (2010). Populations were classified as “undisturbed” if the floristic complexity of vegetation remained relatively intact and the boundary of the population (and the number of plants) was determined by the natural ecological restrictions of the ironstone formation. Populations were described as “disturbed” if surrounding vegetation had been removed or modified thus reducing the population size. In addition, the understory vegetation was classified as either “intact” if it retained the natural floristic array or “degraded” if the native species had been replaced by weedy grasses.

### Sampling for fruit and seed production

As an indicator of relative population fitness, we estimated reproductive output (fruit and seed production) in seven populations that encompassed a range of population sizes and varying vegetation conditions (Table 1). Ten reproductively mature plants were randomly sampled per population where possible. When there were <10 plants, all plants were sampled. To account for seasonal variation, the numbers of fruits and seeds produced per gram branch weight were measured in 2006, 2007, and 2008. On each plant, the branch with the greatest number of fruit and its four closest neighboring branches were harvested. The numbers of green fruit (current season), mature fruit (previous season), and open fruit (>2 years old) were determined and dry‐weight values determined.

**Table 1:** Characteristics of populations of *Hakea oldfieldii* sampled for fruit and seed production

Population	Census size	Population and understory condition^a^	Local remnant vegetation (ha)	Distance to nearest population (m)
IRO	>2000	Undisturbed, intact	443.6	2051
GAL	>1000	Undisturbed, intact	404.6	2098
NEG	>1000	Undisturbed, intact	438.1	3550
PAY	>300	Undisturbed, intact	272.5	2864
KOL	28	Disturbed, intact	615.1	1007
HAI	4	Disturbed, degraded	169.2	6339
COO	2	Disturbed, degraded	16.9	2614

a Descriptions follow Eckert et al. (2010); see Materials and Methods.

Reproductive output was estimated as the mean number of fruit and seed produced per unit branch weight for each plant in each population in each year. In addition, mean seed:fruit ratios were also calculated. We used generalized linear models with randomized complete block designs to examine the effects of population on the fruit and seed production per branch weight and mean seed:fruit ratios. Population was treated as a fixed factor and year as a random blocking factor. Prior to performing statistical analysis, data were tested for normality and homogeneity of variances. The data generally needed square root transformations to stabilize variance, and the results reported in text are back‐transformed. The proportions of sampled plants that produced fruit were averaged for each of the three years. We used Spearman rank correlation coefficients to calculate correlations between population means of (1) fruit production, (2) seed production, and (3) seed:fruit ratios, and census population sizes.

### Sampling for genetic analyses

Mating system and paternity analyses were carried out in three populations (GAL, KOL, PRI; Table 2, Fig. 1). GAL is an undisturbed population (>1000 plants) with intact understory vegetation. KOL is a disturbed population KOL (*N *=* *28) that has been reduced in size by removal of native vegetation, but the local understory vegetation remains intact. PRI is a disturbed population with degraded understory vegetation. These populations were selected within the sampling limitations imposed for threatened species to provide examples of a range of extant populations.

**Table 2:** Mating system and pollen movement parameters estimated for *Hakea oldfieldii* in three populations, GAL, KOL, and PRI

Parameter	GAL	KOL	PRI
Census population size	>1000	28	20
Understory	Intact	Intact	Degraded
Density (plants/ha)	422	101	95
*N*, Total number of adults sampled	102	28	20
Number of families/seed parents sampled	10	11	9
Number of seed sampled	199	210	158
*F*, inbreeding coefficient (SE)	0.031 (0.010)	0.083 (0.015)	0.043 (0.015)
MLTR population mating system estimates			
*t*_m_ (SE), outcrossing rate	0.965 (0.018)	0.971 (0.023)	0.891 (0.053)^a^
*r*_s_ *,* correlation of selfing among loci	0.000 (0.007)	0.000 (0.026)	0.000 (0.020)
*r*_p_ (SE), correlated paternity	0.082 (0.022)^a^	0.199 (0.038)^a^	0.224 (0.072)^a^
1/*r*_p_, mean number of effective pollen parents	12.2	5.0	4.5
Full‐pedigree paternity estimates			
*N*_p_ (SD), mean number of pollen parents/seed parent	9.8 (0.8)^a^	5.6 (0.5)^b^	5.0 (0.58)^b^
Within the sample/population	4.2	4.7	5
Outside the sample/population	5.6	0.9	0
*cp*_div_ (SE), correlated paternal diversity	0.233 (0.059)^c^	0.284 (0.036)^c^	0.486 (0.098)^d^
*FS*_outcross_(SE), mean proportion of outcrosses that are full‐siblings	0.65 (0.12)	0.71 (0.21)	0.69 (0.29)
*r*_ij_ (SE), pollen source overlap	0.016 (0.027)	0.053 (0.033)	0.029 (0.041)
Mean no. of pollen parents siring multiple seed/seed parent (SD)	3.7 (1.49)	2.73 (0.79)	1.89 (1.27)
Total number of pollen donors identified	25	12	11
Number (%) of sampled plants that were pollen parents	13 (12.7%)	10 (35.7%)	11 (55%)
Mean number of seed sired by a pollen parent (SD)	9.4 (1.9)	14.7 (4.2)	13.2 (6.2)
Mean/maximum distance between plants (m)	24.5/67.4	48.1/139.21	58.3/189.4
Mean/maximum distance between nearest neighbors (m)	4.2/26.5	7.2/28.0	14.7/70.3
Mean/maximum pollination distance (m)	9.19/32.4	33.2/131.5	29.3/186.5
Pollinations from <10 m	41.7%	39.1%	37.9%
Pollinations from <20 m	53.3%	58.6%	76.3%
Pollinations from >50 m	42.2%	32.4%	18.4%
Pollen immigration	42.2%^b^	14.8%	0%

Different superscript letters indicate significant difference of estimates (*P *<* *0.05).

a Significantly different from zero, *P *<* *0.05.

b Immigration into sample area within population.

All plants within a 50 m × 70 m sampling area in GAL (*N *=* *102) and all plants in KOL (*N *=* *28) and PRI (*N *=* *20) were mapped, and leaf samples were taken to genotype adult plants. Open‐pollinated fruits were collected from 10 seed parents in GAL, 11 seed parents in KOL, and nine seed parents in PRI. Fruit from seed parents were bulked and desiccated in a drying oven (27°C) to release the seeds, which were germinated and grown in a glasshouse for 1 month. Leaf tissue was harvested from 17 to 21 seedlings per seed parent for DNA extraction and genotyping to give the following totals of genotyped progeny: GAL, 199; KOL, 210; and PRI, 159.

### DNA extraction and genotyping

DNA was extracted from 120 mg of leaf material per plant using a scaled‐down version of the CTAB method (Elliott and Byrne 2005) and genotyped at seven microsatellite loci (HoA102, HoB103, HoB126, HoB010, HoB105, HoA116, and HoB125) using primers and conditions developed for *H. oldfieldii* by Byrne and Hankinson (2009).

Amplification products were separated on a 3730 capillary sequencer (Applied Biosystems, Foster City, CA) using a LIZ 500 (‐250) size standard. Bins were set and genotypes were scored using GENEMAPPER version 4.0 (Applied Biosystems) and checked manually. Samples were repeated for loci that failed to amplify or where seed genotypes were inconsistent with maternal genotypes. In addition, approximately 5% of samples were reamplified and rescored to check scoring accuracy.

To validate the microsatellite data, genotypes were tested for large allele dropout and stuttering artifacts, and the occurrence of null alleles using MICROCHECKER V. 2.2.3 (van Oosterhout et al. 2004). Tests for linkage disequilibrium were performed with global and exact tests for each locus/population combination using GENEPOP 4.2 (Rousset 2008).

### Spatial genetic structure

To test for spatial genetic structure within the sampled populations, we used the kinship coefficient (F*ij*) of Loiselle et al. (1995) that measures the extent of genetic similarity between individuals relative to the mean similarity between individuals. Coefficients were calculated in SPAGeDI v1.2 (Hardy and Vekemans 2002) for distance classes with equal numbers of comparisons. The *S*_*p*_ statistic (Vekemans and Hardy 2004) assesses the rate of decrease in kinship between individuals over distance and, under certain conditions, estimates the reciprocal of neighborhood size. The *S*_*p*_ statistic was calculated for the 1‐ to 10‐m interval.

### Mating system parameters

We used the maximum‐likelihood sibling‐pair method and the correlated mating model implemented in the program MLTR 3.4 (Ritland 2002) to estimate multilocus outcrossing (*t*_m_), the proportion of selfing due to biparental inbreeding (1 − *r*_*s*_), and correlated paternity (*r*_p_), for 10, 11, and nine families in GAL, KOL, and PRI, respectively (Table 2). Estimates were made using seven, seven, and six loci in GAL, KOL, and PRI, respectively, with the null allele option on for loci with frequencies of genotyping errors >0.1. Pollen and ovule gene frequencies were estimated separately. Standard errors were based on 1000 bootstraps.

### Paternity analyses

We used the full‐pedigree likelihood methods implemented in the program COLONY 2.0 (Jones and Wang 2010) to estimate inbreeding coefficients (*F*) and family mating system parameters based on the paternity of seed: the number of outcross pollen parents (*N*_p_), the proportion of outcrossed full‐siblings (*FS*_outcross_), and the correlation of outcross paternal diversity (*cp*_div_). Correlated outcross paternal diversity (*cp*_div_) was calculated as the proportion of outcrossed full‐siblings divided by the number of pollen parents identified among the full‐siblings (Sampson et al. 2014). Paternity analyses for GAL, KOL, and PRI were conducted using multilocus genotypes at seven, six, and six loci, respectively. Analyses used genotypes of adult plants (102, 28, and 20, respectively), seed parents (10, 11, and 9, respectively), and seed (199, 210, and 158, respectively). Estimates of genotyping errors for each locus made using the program Nm + 1.1 (Chybicki and Burczyk 2010) (see Table S1) were included in analyses.

COLONY 2.0 uses a maximum likelihood to assign both sibship and parentage relationships in which all individuals are considered and partitioned simultaneously, resulting in higher power for parentage resolution than is found when pairs of individuals are considered (Wang and Santure 2009; Jones and Wang 2010; Jones et al. 2010). Candidate parents are assigned to clusters of related individuals with 95% confidence. If no candidate parents are available, or if no suitable candidate parents are found, the program will reconstruct parental genotypes. Unlike many other methods of paternity analyses, COLONY implements an error model that simultaneously manages null alleles or allelic dropout (type 1 errors) and other stochastic error (type 2) such as mutation or random typing errors. This model assumes that error occurs independently across loci and the specified error is then incorporated in the group‐likelihood calculations. The full‐pedigree likelihood method of parentage analysis is also little affected by mutation and inbreeding. It is moderately robust to linkage of markers and has been shown to perform well for 3–20 microsatellite loci (Wang and Santure 2009; Jones and Wang 2010; Harrison et al. 2013) and for population samples where it is not possible to confirm that all potential parents have been sampled (Harrison et al. 2013). This is the case for this study and for many others on natural populations (e.g., Scheepens et al. 2012; Saro et al. 2014; Tambarussi et al. 2015). Karaket and Poompuang (2012) have shown that parentage could be completely resolved with four highly informative loci using COLONY when genotyping error was set to 0.05; if genotyping error was set at 0.2, parentage was assigned for 85–90% of offspring with 95% confidence. Error levels for the loci included in this study were below 0.1, and mean estimates of PIC were high to moderate in GAL, KOL, and PRI (0.630, 0.510, and 0.440, respectively). Loci sampled for this study were therefore considered suitable for reliable analyses of paternity using COLONY.

Comparisons of *N*_p_, *FS*_outcross_, and *cp*_div_ among families were made by ANOVA. We used single‐degree‐of‐freedom contrasts to test for association between census population size (large >1000 vs. small <30), or habitat quality (intact vs. degraded understory vegetation), and mean family parameters (*N*_p_, *FS*_outcross_, *cp*_div_).

### Pollen movements within populations and immigration

To estimate pollen movements within sampled populations, we used the paternities inferred from full‐pedigree analyses. We also used the full‐pedigree likelihood method to estimate pollen immigration into the sampled area in GAL, and into the KOL and PRI populations. Pollen immigration rates were estimated as the proportion of the total seeds within each population for which no pollen parent was identified from within the population.

Observations indicate *H. oldfieldii* plants do not flower every year, and we were not able to identify which plants flowered in the years in which the sampled fruit were set. Therefore, to estimate the distance each seed parent was from all potential pollen sources (plant isolation), we calculated the mean distance to all other plants in the population. We tested for correlations between *N*_p_, *FS*_outcross_, and *cp*_div_ and (1) plant isolation and (2) mean pollination distances of each seed parent. To compare the pollination success of each pollen parent (male success), we tested whether numbers of assigned pollinations were correlated with mean pollination distances, and with mean distances to all other plants in the population.

To identify whether seed parents were exposed to the same pollen pool, the degree of pollen source overlap between pairs of seed parents (*r*_ij_) was estimated following Smouse and Robledo‐Arnuncio (2005).

To provide an alternative estimate of pollen dispersal and immigration for comparison, we used the *neighborhood* model in the program Nm + 1.1 (Chybicki and Burczyk 2010) to estimate the proportion of pollen coming from “infinite” *neighborhoods* around seed parents. For KOL and PRI populations where the sample is the entire population, the “infinite” *neighborhood* can be used to estimate contemporary pollen immigration. Pollen dispersal was modeled comparing exponential power and Weibull dispersal kernels to estimate the average distance of dispersal (*d*_p_) and the shape of the dispersal kernel tail (*b*). We excluded loci with genotyping error rates above 0.1 (HoB105 in KOL) and set precision at 0.001.

## Results

### Microsatellite data validation

No evidence of stutter or large allele dropout was found. Two instances of significant linkage disequilibrium were detected among 63 pairwise comparisons in three populations. These findings are unlikely to indicate chromosomal linkage as 3.15 instances were expected by chance from type I error at *P *<* *0.05. Paternity analyses in COLONY are robust to the linkage of markers, and therefore, no loci were excluded on the basis of the observed linkage disequilibrium.

The seven microsatellite markers contained 78 alleles over all populations with from two to 17 alleles per locus (see Table S2). The locus HoA116 was monomorphic in the PRI population and was excluded from analyses in PRI. The mean PIC values for GAL, KOL, and PRI were high to moderate (0.630, 0.510, and 0.440, respectively). General genotyping errors estimated with Nm + 1.1 were relatively low (<0.1), except for locus HoB105 in KOL (see Table S1). This locus was excluded from full‐pedigree analyses for KOL.

### Fruit and seed production

**Figure 2:**
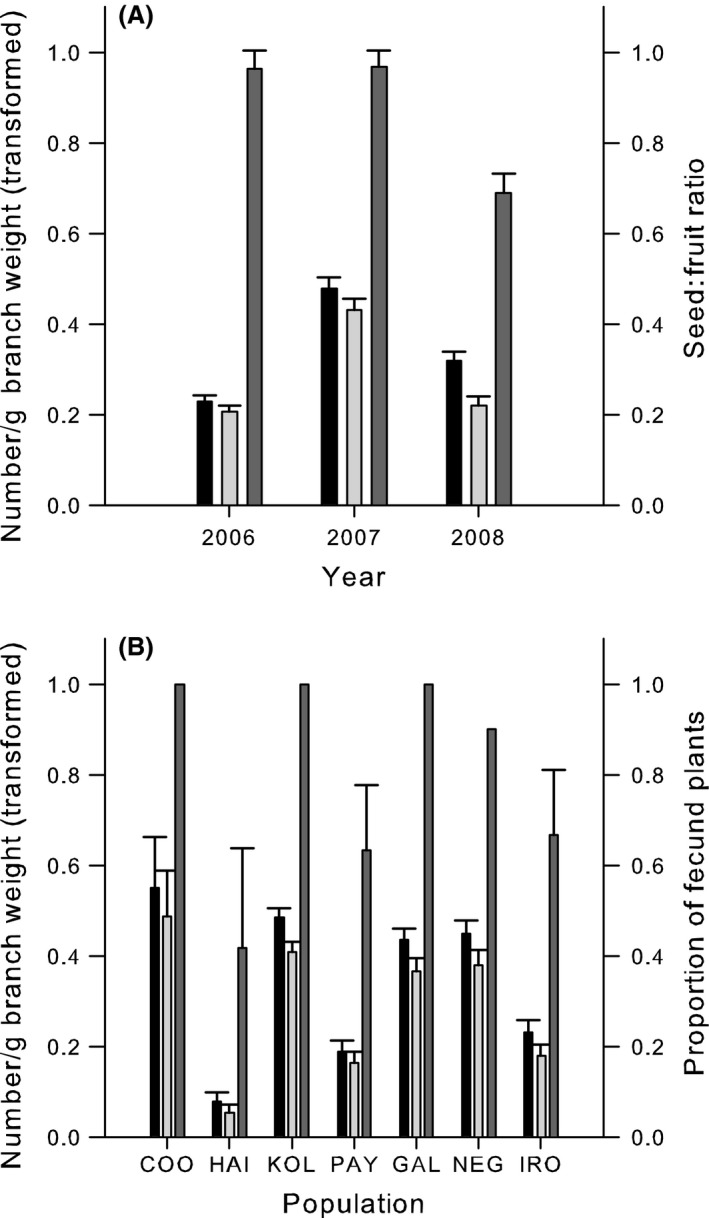
Reproductive outputs of sampled *Hakea oldfieldii* populations. (A) Fruit (black) and seed (light gray) production per gram of branch weight and seed:fruit ratio (dark gray) by year. (B) Fruit (black) and seed (light gray) production per gram of branch weight, and the proportion of sampled plants that were fecund (dark gray). Bars represent standard errors. Populations listed in the order of increasing size. See Table 1 for population codes.

The trends in seed production followed those of fruit production (Fig. 2A). The seed:fruit ratio showed no significant variation between populations (*F*_1,6_ = 0.60, *P *=* *0.73), but there was a significant difference between years (*F*_1,6_ = 13.66, *P *<* *0.0001; Fig. 2A) with 2008 showing a significantly lower seed:fruit ratio (0.69) than 2006 or 2007 (0.96–0.97).

There were significant differences in mean mature fruit production between populations (0.01–0.30 fruit/gm branch weight, *F*_1,6_ = 52.34, *P *<* *0.0001), and in mean seed production between populations (0.01–0.30 fruit/gm branch weight, *F*_1,6_ = 22.45, *P *<* *0.0001). Both fruit production and seed production were significantly lower in HAI (*N *=* *4), PAY (*N *>* *300), and IRO (*N *>* *2000), but the two smallest populations (HAI, COO) showed both the lowest and highest fruit and seed production. The proportion of sampled plants setting fecund fruit varied between populations, but there was also variation between years (Fig. 2B). For example, in two very small populations, the proportion of plants producing fruit and seed ranged from 0 to 75% between years in HAI but was 100% consistently in COO. There were no significant correlations or obvious relationships between fruit or seed production and the census size of populations.

### Spatial genetic structure

There were significant positive correlations between kinship coefficients (*F*_ij_) in the 0 to 10 m distance class in all populations sampled (GAL *F*_ij_ = 0.027, SE = 0.013, *Sp* = 0.019; KOL *F*_ij_ = 0.113, SE = 0.042, *Sp* = 0.065; PRI *F*_ij_ = 0.128, SE = 0.027, *Sp* = 0.058) (see Fig. S1), indicating plants within this distance class were genetically similar. But the kinship value was low in GAL, and only in KOL and PRI did *F*_ij_ values approach those expected for half‐siblings (0.125; Jones and Hubbell 2006). The higher *Sp* values in KOL and PRI suggest neighborhood sizes might be smaller in these populations. But both population density and the mating system have been shown to be correlated with *Sp* by Vekemans and Hardy (2004): *Sp* values were higher in selfing species and less dense populations. The densities of KOL (101 plants/ha; Table 2) and PRI (95 plants/ha) populations were much lower than that of GAL (422 plants/ha).

### Mating system and paternal diversity

Multilocus maximum‐likelihood estimates of outcrossing were high and not significantly different from one in GAL (*t*_m_ = 0.965, SE = 0.018; Table 2) and KOL (*t*_m_ = 0.971, SE = 0.023), and only slightly so in PRI (*t*_m_ = 0.891, SE = 0.053), indicating predominant outcrossing in *H. oldfieldii* (overall mean *t*_m_ = 0.942). Inbreeding coefficients (*F *=* *−0.031 to 0.043; Table 2) were small. Correlation of selfing among loci (*r*_s_) estimates were zero in all populations, suggesting any selfing detected was probably due to biparental mating rather than self‐pollination. This is based on Ritland's (2002) observation that for lower levels of selfing (*s *<* *0.2), *r*_s_ directly approximates the fraction of inbreeding due to self‐pollination. This estimate does not suffer from the bias that affects estimates of biparental inbreeding based on comparison of single‐ and multilocus estimates of *t*. Correlated paternity (*r*_p_ = 0.082–0.224, overall mean 0.168; Table 2) indicated significant proportions of full‐siblings in all populations, equating to moderate neighborhood sizes (1/*r*_p_ = 4.5–12.2, overall mean 5.95). Estimates of *r*_p_ were lowest in the GAL population where there were more potential mates but differences were not significant. Estimates of the overlap of pollen source between seed parents (mean *r*_ij_) were low in all populations (0.016–0.053; Table 2) indicating differences in the pollen pools of seed parents.

**Figure 3:**
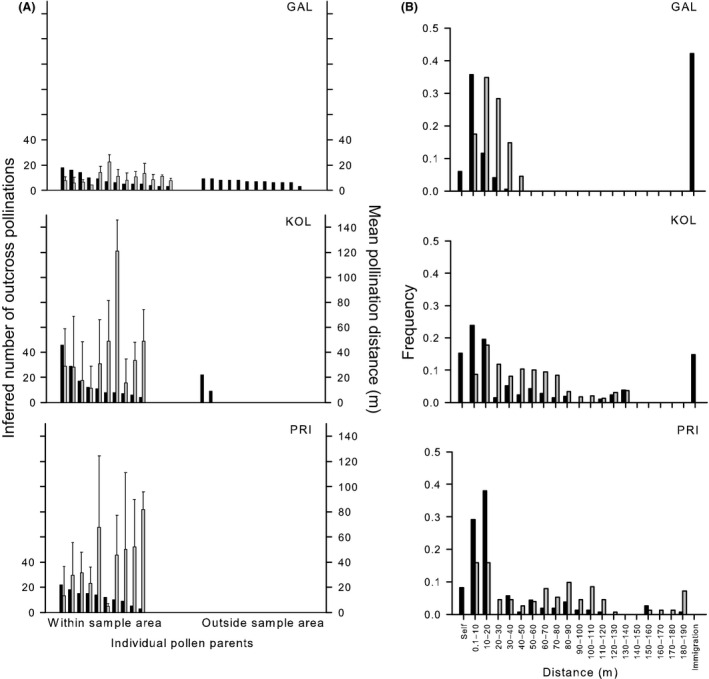
Pollination distances and individual male success in study populations of *Hakea oldfieldii*. (A) For each individual pollen parent, the number of inferred pollinations (black bars) and mean distances of inferred pollinations (gray bars with standard errors). (B) Frequency distribution of inferred pollination distances (black bars) and interplant distances of all plants relative to sampled seed parents (gray bars).

The majority of plants sampled did not sire seeds in GAL and KOL, although 55% did in PRI (Table 2; Fig. 3A), and the number of seed sired by individual pollen parents in GAL was lower than in KOL or PRI.

Overall, families were characterized by large proportions of full‐siblings (*FS*_outcross_; overall mean = 0.686, SE = 0.038), moderate numbers of pollen donors (*N*_p_; overall mean = 6.8, SE = 0.554), and moderate correlation of paternal diversity (*cp*_div_; overall mean = 0.322 SE = 0.040). When the mating parameters of 30 families were compared by ANOVA, there were no significant differences in the proportions of full‐siblings, but the number of pollen donors (*N*_p_, *F*_2,27_ = 4.53, *P *=* *0.020) and the correlation of paternal diversity (*cp*_div_, *F*_2,26_ = 3.82, *P *=* *0.035) did vary significantly. Families from GAL had significantly more outcross pollen parents than families from KOL and PRI (*t *=* *−2.96, *P *=* *0.006). This is not unexpected because there were many more potential mates in the larger GAL population.

When *cp*_div_ was compared among families, it was significantly lower in families from GAL and KOL (*t *=* *−2.72, *P *=* *0.012) than from PRI. GAL and KOL populations differed greatly in size (*N *>* *1000 vs. *N *=* *28, respectively) but have intact understory vegetation. The distribution of paternity was more uneven with fewer pollen parents siring more of the full‐siblings in PRI which has a degraded understory vegetation.

### Pollen movements within populations and immigration

Pollination distances were significantly less than the mean distances between plants but greater than the distances between nearest neighbors in all populations (*P *<* *0.05), indicating pollen movements were not random (Table 2; Fig. 3). The mean pollination distance was lower in GAL (9.19 m; Table 2) than in KOL (33.2 m) and PRI (29.3 m).

Seed parents in all populations had similar proportions of pollinations from sources within 10 m (GAL 41.7%, KOL 39.1%, PRI 37.9%) despite differences in plant density; the density of plants in GAL was four times that of KOL or PRI. However, the majority of pollen came from plants more than 10 m away in all populations (Table 2; Fig. 3B) and there were also significant proportions from more distant plants (>50 m) particularly in GAL. Indeed for GAL, 42.2% of outcross pollen came from outside the sample area (>67 m). The most likely source of this pollen was the hundreds of plants outside the sample area but within the total population. The proportion of successful pollen from sources <20 m away from seed parents was higher in KOL (58.6%) and PRI (76.3%) than in GAL (53.3%), although mean distances between plants were higher in KOL and PRI than in GAL (48.1 m, 58.3 m vs. 24.5 m, respectively; Table 2).

High proportions of pollen came from sources within the population in KOL (85.2%) and PRI (100%), and the maximum pollination distances extended over the maximum possible distance between plants (Fig. 2B). No immigrant pollen was detected in PRI, but analyses suggest immigrant pollen in KOL (14.8%) came from two different pollen parents.

Overall, mean pollination distances of seed parents were not significantly correlated with *N*_p_, *cp*_div_, or *FS*_outcross_. But in GAL, the mean pollination distances of families were significantly positively correlated with the number of pollen parents (*N*_p_; *r *=* *0.689, *P *<* *0.05) and significantly negatively correlated with *cp*_div_ (*r *=* *−0.701, *P *<* *0.05). This suggests that in GAL, seed parents with longer mean pollination distances had more pollen parents and more even paternal diversity.

Overall, there were also no significant correlations between male success (the number of seeds sired by a pollen parent) and mean pollination distances for families or mean distance to other plants. But in PRI, male success was significantly negatively correlated with the pollen parent's mean pollination distances (*r *=* *−0.736, *P *<* *0.05). Male success was not significantly correlated with the mean distances to all other plants in the population (plant isolation). This suggests successful pollination of more distant plants was less likely for pollen parents in this population where the understory vegetation was degraded.

*Neighborhood* model‐based estimates of pollen movements made with NM+ indicated different shaped pollen dispersal in GAL compared to KOL and PRI (Table 3). Pollen dispersal was fat‐tailed (*b *<* *1) in GAL with the highest likelihood found with the Weibull dispersal kernel, although it was only slightly higher than that of the exponential power model. Pollen immigration from outside the sample area was estimated as 46.8%, which was similar to the estimate based on full‐pedigree analyses (42.2%). The estimated shapes of the dispersal curves in KOL and PRI were thin‐tailed indicating less pollen from more distant sources but 19.5% and 14.3% pollen immigration, respectively. These results are higher than those based on full‐pedigree analyses but may be biased because of the linear shape of the populations. Immigration rates estimated using the *neighborhood* model are a function of the dispersal kernel and are based on the assumption that the neighborhood is circular (Goto et al. 2006) resulting in a bias when the populations are linear.

**Table 3:** Pollen immigration and dispersal parameters of the neighborhood model (Chybicki and Burczyk 2010) estimated for an infinite Neighborhood radius in populations of *Hakea oldfieldii*

Population	*s (*SE)	*b* (SE)	*d*_p_ (m)	*m*_p_ (SE)
GAL	0.027 (0.015)	0.694 (0.188)	14.4	0.468 (0.044)
KOL	0.081 (0.029)	1.159 (0.117)	27.0	0.195 (0.057)
PRI	0.121 (0.042)	1.728 (0.075)	38.9	0.143 (0.055)

*s*, selfing rate; *b*, pollen dispersal kernel shape; *d*_p_, mean within neighborhood distance of pollen dispersal; *m*_p,_ pollen immigration.

## Discussion

We found high outcrossing rates in *H. oldfieldii* despite the possibility of geitonogamous pollination among flowers in racemes. Although there were significant correlated matings, the number of pollen parents per seed parent was moderate and the majority of successful pollen came from more distant (>10 m), relatively unrelated sources within populations. High levels of outcrossing have been reported previously in many members of the Proteaceae (Holmes et al. 2009; Krauss et al. 2009; Ayre et al. 2010; Llorens et al. 2011). In *H. oldfieldii*, the correlation of selfing among loci estimates suggested that all the selfing detected was due to biparental mating rather than self‐pollination, suggesting there may be a mechanism to prevent self‐pollination in this species.

In *H. oldfieldii*, there was low but significant spatial structure (0–10 m) around plants in all populations and the significant number of pollinations from within this area would lead to the biparental inbreeding observed. This spatial genetic structure may have developed because seeds are large and heavy with have no adaptations for dispersal and are unlikely to disperse more than a few meters from the maternal plant.

Significant correlated matings and a low proportion of plants that were successful pollen parents might be expected to reduce pollen parent representation in seed crops. However, the number of pollen parents represented among the one or two seasons of seed production represented in the canopy of *H. oldfieldii* (*N*_p_ = 6.8, 1/*r*_p_ = 7.23) was comparable or higher than those reported for other animal‐pollinated plants: *Grevillea iaspicula* (1/*r*_p_ = 2.41; Hoebee and Young 2001); *Pachira quinata* (1/*r*_p_ = 3.97; Quesada et al. 2001); and *Banksia sphaerocarpa* var. *caesia* (1/*r*_p_ = 2.98–7.04; Llorens et al. 2011). The canopy‐stored seed, upon which the species relies for survival following fires, would therefore contain moderate genetic diversity because of the combined effects of high outcrossing rates and moderate paternal diversity.

The high proportion of full‐siblings, the low overall number of pollen parents, and the differences in pollen pools among seed parents that we observed here in *H. oldfieldii* populations could be due to a sparse distribution of flowering plants, variation in flowering phenology, and/or pollinator behavior. But these factors would not explain the low selfing observed in *H. oldfieldii*. Population size, plant density, isolation, and spatial structure are known to affect the mating system in plants (Young et al. 1996; Sork et al. 1999; Hoebee et al. 2007; Duminil et al. 2009), but in *H. oldfieldii* outcrossing rates did not differ significantly among populations of different size, shape, or plant density. We suggest our findings are consistent with the operation of a self‐incompatibility (SI) system in *H. oldfieldii*. Formal confirmation of SI would require controlled crossing experiments that were not possible in this study. Gametophytic SI (controlled by the gametophyte) is the only form of SI that has been reported in the Proteaceae and, as incompatibility systems tend to be conserved within families (Hoebee and Young 2001), it would be the type likely to occur in *H. oldfieldii*. We detected low levels of inbreeding in *H. oldfieldii*, but these do not exclude the possibility of an SI system. Quantitative variation in the strength of SI responses is a recognized phenomenon (Busch and Schoen 2008), and some self‐fertilization or mating between relatives can occur. We suggest therefore that it may be the distribution of compatible mates throughout the population that results in a large proportion of successful pollen coming from more distant and unrelated sources. The combination of high outcrossing and pollen dispersal outside the area of significant spatial structure within populations would promote paternal diversity among seeds. Such diversity is selectively advantageous (Pannell and Labouche 2013) and would be particularly important in a species that relies on the small amounts of canopy‐stored seed for survival following destruction of populations.

Gametophytic SI in *H. oldfieldii* could also explain the variable seed and fruit production found among populations, and the contrasting seed and fruit production of different small populations. In gametophytic incompatibility, seed and fruit production need not be associated with population size but should be maintained in populations as long as the population size remains above a threshold where the availability of compatible genotypes becomes limiting (Pickup and Young 2008). However, the significant temporal variation in fruit and seed production between years that was also observed for some populations is difficult to explain. It was not clearly related to climatic data, but this lack of correlation may be a result of a lack of knowledge of the critical times in fruit development. In addition, the causes of significant reduction in seed to fruit ratio between years were unclear.

### Pollen dispersal and paternal diversity

The pollen dispersal patterns found in *H. oldfieldii* were leptokurtic, with a higher frequency of short distance pollinations. This is the most common pattern found in plants (Ashley 2010), and our observations are also consistent with the pattern frequently reported for insect‐pollinated species (Austerlitz et al. 2004; Smouse and Sork 2004; Vekemans and Hardy 2004). But in *H. oldfieldii*, most pollen movements were not to nearest neighbors but to plants >10 m away, including significant pollination from distant (>50 m away) sources. This pattern of pollen dispersal would promote paternal diversity among seeds because significant spatial structure was low and only found up to 10 m around plants. We propose that the diversity of paternity observed within families in *H. oldfieldii* is promoted by a combination of short and distant pollen movements to the limited number of mates spread throughout the population, although the direct relationship between these factors was not able to be determined by this study. The lack of seed production of individual plants in some years, the high proportions of pollen parents that were also seed parents, the low overlap in the pollen sources of seed parents, and the lack of pollination success of some individuals could be the result of a number of factors, including mate limitation due to SI, lack of floral initiation in some plants, or a mismatch between flowering and the availability of pollinators.

### Pollen immigration

As Ashley (2010) noted when reviewing pollination studies, mating patterns are influenced by a complex array of factors such as compatibility, flowering phenology, pollinator abundance, and behavior. Significant pollen dispersal within populations may not equate to significant dispersal between disjunct populations because the conditions for pollen movement and/or the array and behavior of pollinators may be different in different habitats. Full‐pedigree analyses detected some pollen immigration (14.8%) into one of the two small populations for which all plants were sampled. As this population and the surrounding area were thoroughly searched for *H. oldfieldii* plants, it is unlikely the pollen came from an undetected local plant. The nearest population was over 1 km away and therefore this provides evidence for some pollen dispersal between populations across different habitat at a level similar to those found in other woody species with disjunct populations (Robledo‐Arnuncio and Gill 2005; Hoebee et al. 2007; Sampson et al. 2015). However, the level was relatively low when compared to a range of woody species reviewed by Ashley (2010) and Ellstrand (2014). High pollen immigration (42.2%) into the sample area within the large GAL population was most likely to be from the >1000 plants in the surrounding population. Estimates of pollen immigration made into KOL and PRI using the *neighborhood* model were higher (14.3–19.5%) than direct estimates based on paternity. But estimates are of a similar magnitude when the bias introduced by the model assumption of circular neighborhoods around plants was taken into account. However, pollen immigration rates do not measure effective gene flow, that is, where genes become established in a population and subject to the impact of evolutionary forces (Ellstrand 2014). Direct pollen immigration estimates in the range of 0–14.8% are therefore not inconsistent with the low gene flow (*N*_m_ < 1 Sampson et al. 2015) estimated for 14 *H. oldfieldii* populations using indirect methods. If pollen immigration is low, we suggest that high outcrossing rates and pollen dispersal within populations would be important contributors to paternal and genetic diversity among seeds in *H. oldfieldii*.

### Mating patterns in degraded habitat

We found some indications that mating patterns in *H. oldfieldii* may be affected by the condition of the understory vegetation but our study included only one population with degraded understory vegetation. Inferences about mating patterns and their association with ecological parameters such as distance, density, flowering and fragmentation are often reported in the literature based on single populations or multiple populations of different size, condition and level of genetic isolation (Goto et al. 2006; Geng et al. 2008; Llaurens et al. 2008; Ottewell et al. 2012; Scheepens et al. 2012; Tambarussi et al. 2015; Medina‐Macedo et al. 2016). This is particularly common for rare or threatened species because of the practical and ethical issues limiting sampling. But inferences about mating patterns and ecological variables arising from these studies are nevertheless worth making: firstly, because they add to the body of knowledge and may be used in combination with other studies to reveal patterns, and secondly, because they suggest directions for future research.

With these limitations in mind, we found families with degraded understory vegetation showed higher correlation of paternal diversity when compared to families with an intact understory, regardless of whether they were from small or large populations. In addition, significantly more male success was associated with shorter pollination distances when the understory vegetation was degraded. Previous studies have shown that the behavior of pollinators within degraded remnants is likely to differ from that in vegetation remnants that are in good condition (Yates et al. 2007). Pollinators may be less likely to visit more distant plants in populations where there is little or no native flora between individuals of the target species (see review of Eckert et al. 2010). Increased visits, or time spent on plants because of a lack of alternatives in the depauperate understory, could cause reduction in the numbers of pollen parents and increased correlation of paternal diversity. Supporting this proposition, the highest proportion of pollinations from <20 m occurred in the population with a degraded understory even though plants were further apart on average.

The importance of habitat quality in determining mating patterns has been observed in other plant species where increased inbreeding and reduced paternal diversity have been associated previously with degraded habitat (Coates et al. 2007; Yates et al. 2007; Eckert et al. 2010; Llorens et al. 2011). In naturally disjunct and insular species, high outcrossing rates and variable seed production among populations may indicate more about the evolutionary and demographic history of the species than the impact of recent fragmentation. However, changes in mating patterns in fragmented populations where the understory vegetation has been removed suggest changes in processes that maintain diversity. Protection and restoration of habitat may therefore need to be a priority in conservation planning for species that have evolved with naturally disjunct populations. Further study of replicated populations of different sizes but with degraded verses intact understory vegetation is needed to examine this proposal.

## Conclusions

We found high outcrossing with some biparental inbreeding in *H. oldfieldii* despite the possibility of geitonogamous pollination within inflorescences, suggesting there may be a mechanism to avoid self‐pollination as found in other Proteaceae. Although correlated matings were significant, and the number of successful pollen parents were low within populations, moderate numbers and diversity of pollen parents were maintained for seed parents by a combination of low spatial genetic structure and substantial pollen dispersal. High outcrossing with moderate paternal diversity would help maintain genetic diversity among the relatively small store of seeds in the canopy even when populations become very small following fires that are common in the landscape. These features of the mating system would contribute to the persistence of *H. oldfieldii* as isolated populations.

## Conflict of Interest

None declared.

## Supporting information


**Figure S1.** Spatial autocorrelation coefficients in study populations.

**Table S1.** Estimates of genotyping error calculated in NM + 1.1 (Chybicki and Burczyk 2010) at microsatellite loci in *Hakea oldfieldii* populations for which mating system parameters and pollen‐mediated gene dispersal and immigration were estimated.

**Table S2.** Allele frequencies for microsatellite markers in *Hakea oldfieldii* populations for which mating system parameters and pollen‐mediated gene dispersal and immigration were estimated.
