# Supplementary material for: Limiting inbreeding in disjunct and isolated populations of a woody shrub
Source: Ecol Evol. 2016 Jul 25;6(16):5867–80. doi: 10.1002/ece3.2322 (PMC4983598; doi:10.1002/ece3.2322)
Supplement: Supplementary file 2 — Table S1. Estimates of genotyping error calculated in NM + 1.1 (Chybicki and Burczyk 2010) at microsatellite loci in Hakea oldfieldii populations for which mating system parameters and pollen‐mediated gene dispersal and immigration were estimated. [file ECE3-6-5867-s002.doc]

**Table S1.** Estimates of genotyping error calculated in nm+1.1 (Chybicki and Burczyk, 2010) at microsatellite loci in *Hakea oldfieldii* populations for which mating system parameters, and pollen-mediated gene dispersal and immigration were estimated.

| Population | Locus | Error |
| --- | --- | --- |
|  |  |  |
| GAL | HoA102 | 0.009 |
|  | HoB103 | 0.002 |
|  | HoB126 | 0.000 |
|  | HoB010 | 0.000 |
|  | HoB105 | 0.034 |
|  | HoA116 | 0.012 |
|  | HoB125 | 0.007 |
|  |  |  |
| KOL | HoA102 | 0.048 |
|  | HoB103 | 0.012 |
|  | HoB126 | 0.080 |
|  | HoB010 | 0.024 |
|  | HoB105* | 0.202 |
|  | HoA116 | 0.044 |
|  | HoB125 | 0.019 |
|  |  |  |
| PRI | HoA102 | 0.065 |
|  | HoB103 | 0.092 |
|  | HoB126 | 0.017 |
|  | HoB010 | 0.045 |
|  | HoB105 | 0.000 |
|  | HoA116 | 0.000 |
|  | HoB125 | 0.048 |

* Excluded from analyses
