# Supplementary material for: Limiting inbreeding in disjunct and isolated populations of a woody shrub
Source: Ecol Evol. 2016 Jul 25;6(16):5867–80. doi: 10.1002/ece3.2322 (PMC4983598; doi:10.1002/ece3.2322)
Supplement: Supplementary file 3 — Table S2. Allele frequencies for microsatellite markers in Hakea oldfieldii populations for which mating system parameters and pollen‐mediated gene dispersal and immigration were estimated. [file ECE3-6-5867-s003.doc]

**Table S2.** Allele frequencies for microsatellite markers in *Hakea oldfieldii* populations for which mating system parameters, and pollen-mediated gene dispersal and immigration were estimated

| Locus | Allele | GAL | KOL | PRI |
| --- | --- | --- | --- | --- |
| HoA102 | 222 | 0.381 | 0.339 | 0.475 |
|  | 230 | 0.119 | 0.125 | 0.000 |
|  | 233 | 0.094 | 0.000 | 0.025 |
|  | 239 | 0.015 | 0.000 | 0.000 |
|  | 244 | 0.010 | 0.000 | 0.000 |
|  | 245 | 0.059 | 0.000 | 0.000 |
|  | 247 | 0.312 | 0.161 | 0.000 |
|  | 251 | 0.010 | 0.000 | 0.050 |
|  | 259 | 0.000 | 0.375 | 0.450 |
|  |  |  |  |  |
| HoB103 | 244 | 0.010 | 0.000 | 0.184 |
|  | 246 | 0.000 | 0.000 | 0.026 |
|  | 250 | 0.005 | 0.000 | 0.000 |
|  | 254 | 0.369 | 0.411 | 0.684 |
|  | 256 | 0.242 | 0.000 | 0.000 |
|  | 262 | 0.066 | 0.000 | 0.000 |
|  | 263 | 0.086 | 0.250 | 0.026 |
|  | 265 | 0.005 | 0.000 | 0.000 |
|  | 271 | 0.116 | 0.000 | 0.000 |
|  | 274 | 0.066 | 0.000 | 0.000 |
|  | 275 | 0.005 | 0.000 | 0.000 |
|  | 277 | 0.000 | 0.018 | 0.000 |
|  | 279 | 0.000 | 0.018 | 0.000 |
|  | 287 | 0.000 | 0.304 | 0.079 |
|  | 289 | 0.030 | 0.000 | 0.000 |
|  |  |  |  |  |
| HoB126 | 160 | 0.050 | 0.000 | 0.000 |
|  | 164 | 0.020 | 0.037 | 0.575 |
|  | 166 | 0.015 | 0.000 | 0.000 |
|  | 168 | 0.144 | 0.000 | 0.025 |
|  | 174 | 0.371 | 0.148 | 0.000 |
|  | 176 | 0.045 | 0.000 | 0.000 |
|  | 178 | 0.000 | 0.296 | 0.025 |
|  | 180 | 0.000 | 0.019 | 0.000 |
|  | 182 | 0.050 | 0.000 | 0.000 |
|  | 184 | 0.015 | 0.463 | 0.300 |
|  | 186 | 0.000 | 0.037 | 0.075 |
|  | 189 | 0.020 | 0.000 | 0.000 |
|  | 195 | 0.025 | 0.000 | 0.000 |
|  | 196 | 0.050 | 0.000 | 0.000 |
|  | 197 | 0.178 | 0.000 | 0.000 |
|  | 201 | 0.015 | 0.000 | 0.000 |
|  | 207 | 0.005 | 0.000 | 0.000 |
|  |  |  |  |  |
| HoB010 | 148 | 0.338 | 0.000 | 0.105 |
|  | 150 | 0.025 | 0.000 | 0.000 |
|  | 152 | 0.000 | 0.000 | 0.079 |
|  | 158 | 0.049 | 0.125 | 0.000 |
|  | 160 | 0.108 | 0.036 | 0.000 |
|  | 164 | 0.054 | 0.000 | 0.053 |
|  | 166 | 0.108 | 0.839 | 0.000 |
|  | 169 | 0.015 | 0.000 | 0.000 |
|  | 170 | 0.000 | 0.000 | 0.737 |
|  | 175 | 0.304 | 0.000 | 0.000 |
|  | 183 | 0.000 | 0.000 | 0.026 |
|  |  |  |  |  |
| HoB105 | 204 | 0.045 | 0.107 | 0.325 |
|  | 206 | 0.405 | 0.304 | 0.000 |
|  | 207 | 0.000 | 0.000 | 0.025 |
|  | 208 | 0.010 | 0.357 | 0.300 |
|  | 210 | 0.040 | 0.000 | 0.000 |
|  | 212 | 0.320 | 0.214 | 0.000 |
|  | 213 | 0.015 | 0.018 | 0.350 |
|  | 214 | 0.010 | 0.000 | 0.000 |
|  | 216 | 0.155 | 0.000 | 0.000 |
|  |  |  |  |  |
| HoA116 | 261 | 0.922 | 0.722 | 1.000 |
|  | 288 | 0.078 | 0.278 | 0.000 |
|  |  |  |  |  |
| HoB125 | 281 | 0.000 | 0.000 | 0.200 |
|  | 287 | 0.035 | 0.000 | 0.000 |
|  | 291 | 0.025 | 0.107 | 0.000 |
|  | 295 | 0.056 | 0.000 | 0.000 |
|  | 296 | 0.005 | 0.000 | 0.000 |
|  | 297 | 0.429 | 0.036 | 0.025 |
|  | 299 | 0.146 | 0.411 | 0.150 |
|  | 301 | 0.157 | 0.304 | 0.525 |
|  | 303 | 0.091 | 0.143 | 0.000 |
|  | 305 | 0.025 | 0.000 | 0.000 |
|  | 308 | 0.000 | 0.000 | 0.075 |
|  | 309 | 0.000 | 0.000 | 0.025 |
|  | 310 | 0.005 | 0.000 | 0.000 |
|  | 313 | 0.015 | 0.000 | 0.000 |
|  | 315 | 0.010 | 0.000 | 0.000 |
